# Data analysis for investigating the tribological behaviors of aluminum-silicon alloys

**DOI:** 10.1016/j.dib.2022.108260

**Published:** 2022-05-11

**Authors:** Mahboobeh Azadi, Mohammad Azadi

**Affiliations:** a Faculty of Materials and Metallurgical Engineering, Semnan University, Semnan, Iran; b Faculty of Mechanical Engineering, Semnan University, Semnan, Iran

**Keywords:** Wear dataset, Nanoparticles, Hardness, Elastic module, Nanocomposite, Aluminum alloys

## Abstract

This dataset describes the tribological properties of aluminum-silicon alloys when reinforced with two types of nanoparticles: silica and clay. Moreover, two types of aluminum-silicon alloys were chosen as matrices. These alloys are used in the piston and cylinder-head parts of an automobile engine part. The percentage of nanoparticles was about 1 wt. %. All specimens were developed through the stir casting method. The range of casting temperature was about 700−850 °C. Stirring times for aluminum alloy melts were 2 and 4 min. Ageing heat treatment was also applied for some specimens after the casting process. The Vickers hardness instrument, a pin-on-disk tribometer, and compression test were utilized to collect the data. In addition, to analyze data, a Design-Expert software was used. The data contain hardness, wear rate, friction coefficient, and compression elastic module for aluminum-silicon alloy specimens.

## Specifications Table

**Table:** 

Subject	Engineering
Specific subject area	Engineering/ Materials Engineering/ Manufacturing Engineering/ Automotive Engineering
Type of data	Table and Figure
How the data were acquired	At first, specimens were manufactured through a stir casting method. Then, a pin-on-disk tribometer was used to measure the friction coefficient and wear rate of specimens. A Vickers hardness tester was utilized for measuring the hardness of specimens. A compression test was also applied to report the elastic module. At last, data were analyzed by the regression model to figure out the effects of stirring time and temperature, type of nanoparticles, and ageing heat treatment.
Data format	Raw data and Analyzed
Description of data collection	The wear force and distance: 7 N and 500 m The rotation speed in wear testing: 200 rpm The steel pin hardness in wear testing: 778 HV The speed in compression testing: 1 mm min^−1^ Adding nanoparticles: 0 and 1 wt.% The reinforcing condition: with and without ageing heat-treatment. Test temperature: 20−25 °C
Data source location	Institution: Faculty of Materials and Metallurgical Engineering, Semnan University City/Town/Region: Semnan Country: Iran Latitude and longitude (and GPS coordinates, if possible) for collected samples/data: 35.60645359387411, 53.43099444360283
Data accessibility	Repository name: Mendeley Data Data identification number (permanent identifier, i.e., DOI number): 10.17632/x72b3zgrrf.2 Direct link to the dataset: https://data.mendeley.com/datasets/x72b3zgrrf/2

## Value of the Data

• Utilizing aluminum-silicon alloys in various industries is widespread nowadays. Therefore, measuring material properties is useful for engineers to choose the best material with better characterizations.
• Predicting the effects of various manufacturing parameters on the behavior of the material can limit the manufacturing cost of engineering parts.
• The purpose of the analyzed data presented in this work is to show the effects of stirring time, casting temperature, type of nanoparticles, and ageing heat treatment on tribological behavior of two different reinforced aluminum-silicon alloys. These alloys are used in the piston and cylinder-head parts of an automobile engine part.
• Valuable analysis of mechanical data could be helpful for other materials engineers to know which nanocomposite manufacturing parameter was the most effective one to have higher hardness and elastic module and lower friction coefficient and wear rate.
• These data can be re-used for other researchers to compare their data with the presented data to develop the best aluminum-silicon matrix nanocomposite.

## 1 Data Description

**Fig. 1:**
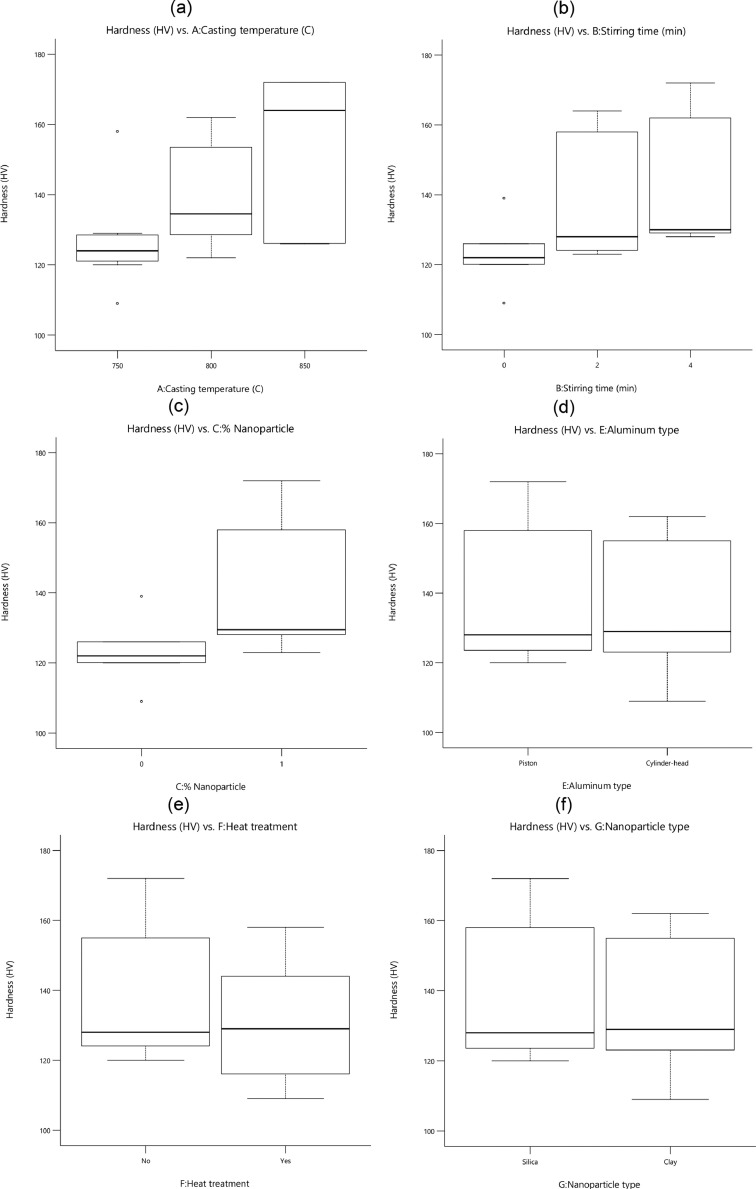
The value of the hardness for different parameters: (a) the casting temperature, (b) the stirring time, (c) the percentage of nanoparticles, (d) the aluminum type, (e) the ageing heat treatment, and (f) the nanoparticle type

Fig. 1 shows the variation and the scatter-band of each utilized parameter versus the hardness. The higher casting temperature and stirring time as casting parameters and adding reinforcements caused an increase in the hardness of specimens. Adding nanoparticles was effective to increase the hardness of the aluminum-silicon matrix. However, the types of nanoparticles and aluminum alloys had no significant influence on the hardness changes of manufactured specimens. Based on the values of the data scatter-band, it was found that the piston aluminum alloy had higher hardness compared to cylinder-head aluminum alloy. Moreover, the silica (SiO_2_) nanoparticles caused higher hardness for nanocomposites compared to clay nanoparticles.

**Fig. 2:**
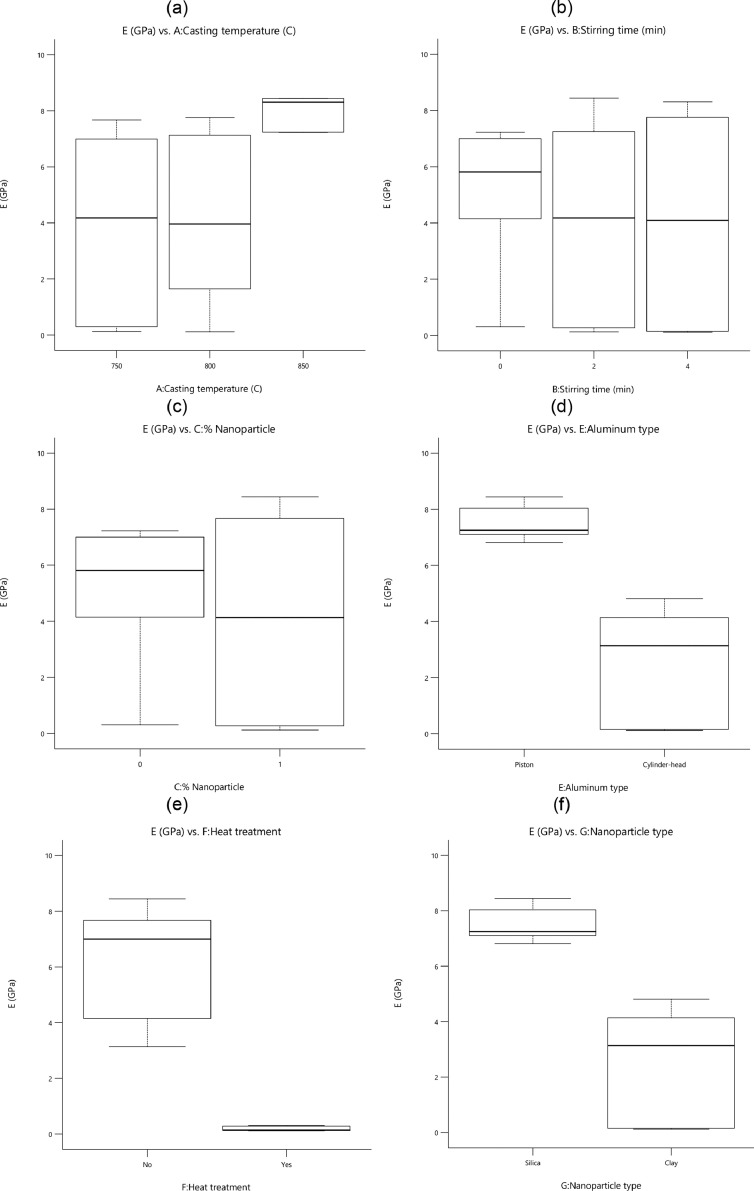
The value of the elastic module for different parameters: (a) the casting temperature, (b) the stirring time, (c) the percentage of nanoparticles, (d) the aluminum type, (e) the heat treatment, and (f) the nanoparticle type

Fig. 2 depicts the effect of each variable on the variation and the scatter-band of the elastic module (*E*). The higher casting temperature and lower stirring time raised the elastic module of nanocomposites. By comparing the scatter-band of *E* values for nanocomposites with respect to the aluminum alloys, the presence of nanoparticles in 1 % wt., had an insignificant effect on increasing this property. The presence of SiO_2_ nanoparticles to increase the elastic module of manufactured nanocomposites was more effective than the other reinforcement. However, the piston aluminum alloy without ageing heat treatment and reinforcement had a higher value of *E* compared to other specimens.

**Fig. 3:**
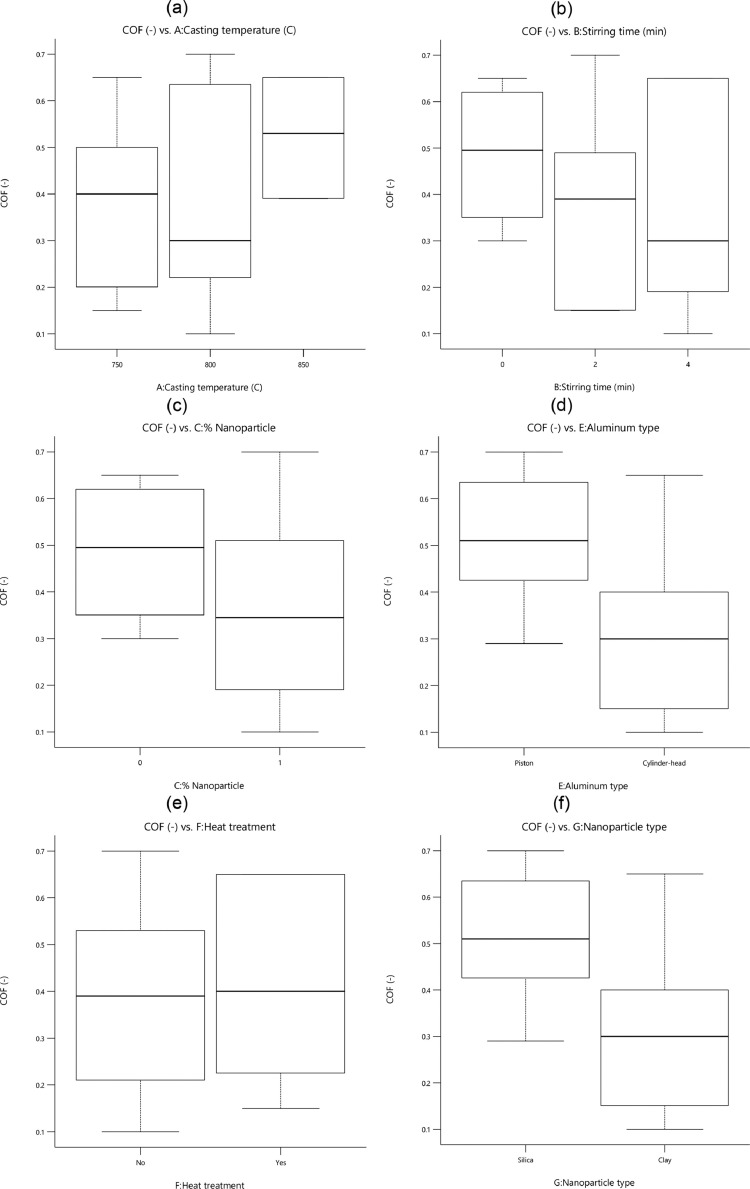
The value of the friction coefficient for different parameters: (a) the casting temperature, (b) the stirring time, (c) the percentage of nanoparticles, (d) the aluminum type, (e) the heat treatment, and (f) the nanoparticle type

Fig. 3 describes the influence of introduced variables on the value of the friction coefficient or the coefficient of friction (COF). The lower stirring time in clay-nanocomposite caused a reduction in values COF. The heat treatment was not an effective parameter to decrease the friction coefficient. Moreover, COF values of cylinder-head aluminum alloy were lower than values for the other utilized alloy. Nanocomposites with clay nanoparticles showed lower COF than other composites.

**Fig. 4:**
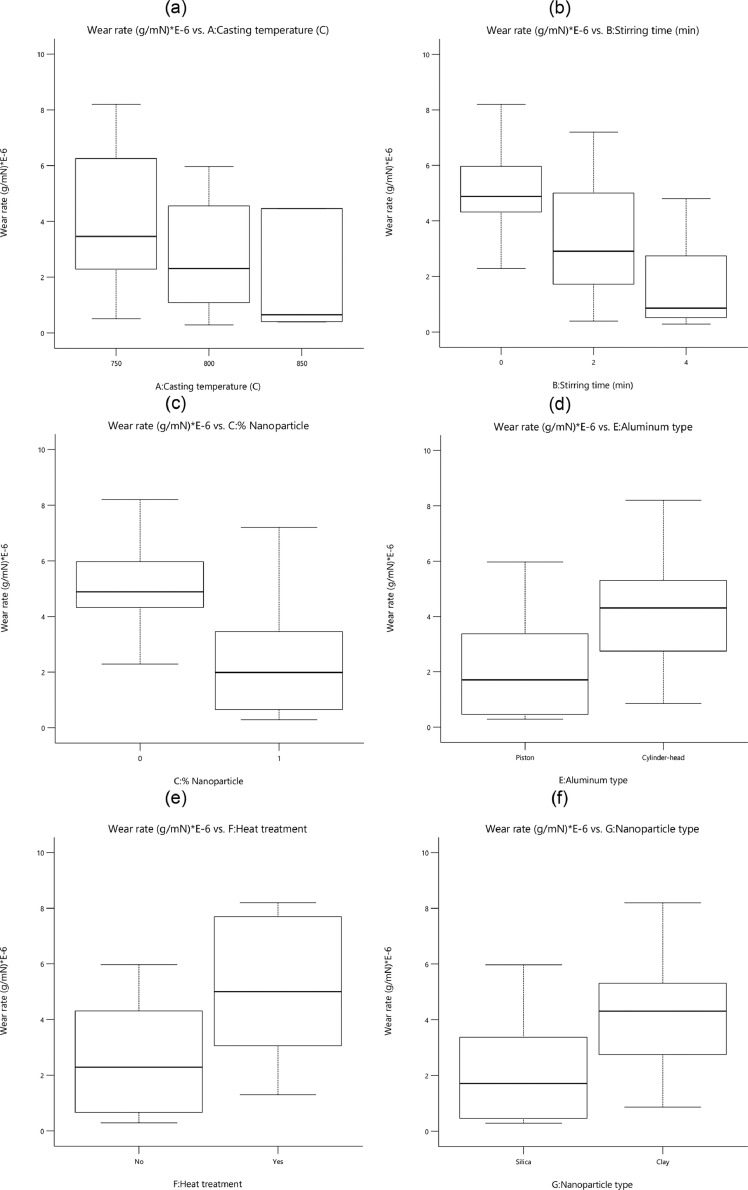
The value of the wear rate for different parameters: (a) the casting temperature, (b) the stirring time, (c) the percentage of nanoparticles, (d) the aluminum type, (e) the heat treatment, and (f) the nanoparticle type

The wear rate versus all variables for all specimens is shown in Fig. 4. The lower stirring time and casting temperature reduced the wear rate for nanocomposites. When nanoparticles were added to the aluminum-silicon matrix the wear rate would be decreased. In addition, the piston aluminum alloy had a lower wear rate compared to cylinder-head aluminum alloy. The heat treatment was not an effective process to reduce the wear rate of specimens. More details about the relation between hardness and wear rate were found in other research [1].

Both qualitative and quantitative analyzes are reported on presented tribological data based on the regression model. In Tables 1, 2, 3, and 4, the results of the regression analysis are reported, using the Design-Expert software. This regression analysis was significant for all outputs (the hardness, *E*, COF, and wear rate), which the P-Value was lower than 0.05 and with the coefficient of determination (R^2^) of 58.25%, 97.83%, 56.17%, and 78.58%, respectively. The adjusted and predicted R² values were lower than R^2^.

**Table 1:** The analyzed data with the regression model for the hardness.

Source	Sum of Squares	DF	Mean Square	F-Value	P-Value	Effect	Score
Model	3517.16	5	703.43	3.91	0.0200	significant	-
A-Casting temperature (C)	1633.75	1	1633.75	9.07	0.0093	significant	1
B-Stirring time (min)	0.59	1	0.59	0.00	0.9550	not significant	-
C-% Nanoparticle	398.06	1	398.06	2.21	0.1593	not significant	-
E-Aluminum type	195.80	1	195.80	1.09	0.3148	not significant	-
F-Heat treatment	246.06	1	246.06	1.37	0.2620	not significant	-
G-Nanoparticle type	0.00	0	-	-	-	not considered	-
Lack of Fit	1908.39	12	159.03	0.52	0.8120	not significant	-
R^2^	58.25%	-					
Adjusted R^2^	43.33%	-					
Predicted R^2^	16.96%	-

**Table 2:** The analyzed data with the regression model for the elastic module.

Source	Sum of Squares	DF	Mean Square	F-Value	P-Value	Effect	Score
Model	175.88	5	35.18	125.97	< 0.0001	significant	-
A-Casting temperature (C)	0.16	1	0.16	0.56	0.4670	not significant	-
B-Stirring time (min)	0.01	1	0.01	0.02	0.8939	not significant	-
C-% Nanoparticle	0.03	1	0.03	0.11	0.7476	not significant	-
E-Aluminum type	38.10	1	38.10	136.43	< 0.0001	significant	2
F-Heat treatment	39.45	1	39.45	141.28	< 0.0001	significant	1
G-Nanoparticle type	0.00	0	-	-	-	not considered	-
Lack of Fit	3.90	12	0.33	75.16	0.0132	significant	-
R^2^	97.83%	-					
Adjusted R^2^	97.05%	-					
Predicted R^2^	95.53%	-

**Table 3:** The analyzed data with the regression model for the friction coefficient.

Source	Sum of Squares	DF	Mean Square	F-Value	P-Value	Effect	Score
Model	0.39	5	0.08	3.59	0.0268	significant	-
A-Casting temperature (C)	0.00	1	0.00	0.08	0.7832	not significant	-
B-Stirring time (min)	0.00	1	0.00	0.09	0.7648	not significant	-
C-% Nanoparticle	0.03	1	0.03	1.29	0.2746	not significant	-
E-Aluminum type	0.28	1	0.28	12.70	0.0031	significant	1
F-Heat treatment	0.14	1	0.14	6.58	0.0225	significant	2
G-Nanoparticle type	0.00	0	-	-	-	not considered	-
Lack of Fit	0.21	12	0.02	0.38	0.8855	not significant	-
R^2^	56.17%	-					
Adjusted R^2^	40.52%	-					
Predicted R^2^	14.85%	-

**Table 4:** The analyzed data with the regression model for the wear rate.

Source	Sum of Squares	DF	Mean Square	F-Value	P-Value	Effect	Score
Model	82.66	5	16.53	10.27	0.0003	significant	-
A-Casting temperature (C)	0.9959	1	0.9959	0.6189	0.4446	not significant	-
B-Stirring time (min)	8.51	1	8.51	5.29	0.0374	significant	2
C-% Nanoparticle	0.4273	1	0.4273	0.2655	0.6144	not significant	-
E-Aluminum type	3.47	1	3.47	2.15	0.1643	not significant	-
F-Heat treatment	15.47	1	15.47	9.61	0.0078	significant	1
G-Nanoparticle type	0.0000	0	-	-	-	not considered	-
Lack of Fit	13.98	12	1.17	0.2727	0.9428	not significant	-
R^2^	78.58%	-					
Adjusted R^2^	70.93%	-					
Predicted R^2^	56.27%						

Notably, in the Design-Expert software, the factor coding was selected as the coded one and the summation of squares was Type 3 - Partial. For the hardness, the model F-value of 3.91 implied the model was significant. This value for *E*, COF, and the wear rate was 125.97, 3.59, and 10.27, respectively. As another note, the parameter of nanoparticle type was not considered, which was aliased.

For more details of the regression analysis, it should be mentioned that for the hardness, the predicted R² of 16.96% was not as close to the adjusted R² of 43.33%, as one might normally expect; i.e., the difference was more than 0.2. This may indicate a large block effect or a possible problem with the model and/or data. The adequate precision has measured the signal to noise ratio, which greater than 4 was desirable. This ratio of 6.562 for the hardness indicated an adequate signal.

For the *E* value, the predicted R² of 95.53% was in reasonable agreement with the adjusted R² of 97.05%; i.e., the difference was less than 0.2. The ratio of signal to noise was 26.449, which demonstrated an adequate signal, as it was more 4. Moreover, the lack of fits was also significant, which should be not significant for proper regression analysis. Therefore, this model is ignored for the elastic module.

For the COF value, the predicted R² of 14.85% was not as close to the adjusted R² of 40.52%, as one might normally expect; i.e., the difference was more than 0.2. The adequate precision was also measured as 5.626, which was proper.

Finally, for the wear rate, the predicted R² of 56.27% was in reasonable agreement with the adjusted R² of 70.93%, less than 0.2. The ratio of signal to noise was also more than 4, as calculated 11.389.

Based on tribological data in Tables 1, 2, 3, and 4, the hardness, COF, and the wear rate could be significantly affected by variables. However, changes in the value of *E* did not change significantly by chosen variables. The most effective parameter to increase the hardness was the stirring temperature for specimens. For changing COF of specimens, the aluminum type and ageing heat treatment were two factors that had effective roles. For the wear rate, the heat treatment process and the stirring time were variables that had the highest effects.

The regression model for the hardness (*H*V), *E*, COF, and the wear rate (*WR*) could be written according to the variables including the casting temperature (A), the stirring time (B), the percentage of nanoparticles (C), the aluminum type (E), and the heat treatment (F), as follows,

(1) HV=133.50+13.97A−0.42B+9.45C+3.88E−4.79F

(2) E=3.86+0.14A−0.04B+0.08C−1.71E−1.92F

(3) COF=0.51+0.01A+0.02B−0.08C−0.15E+0.12F

(4) WR=3.88−0.34A−1.59F−0.31C+0.52E+1.20F

**Fig. 5:**
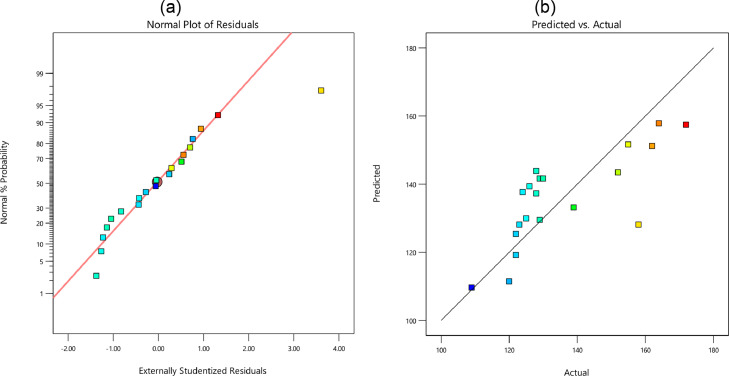
The hardness: (a) the normal plot of residuals and (b) the variations of the predicted and experimental data; Note: Color points are from blue (109) to red (172)

**Fig. 6:**
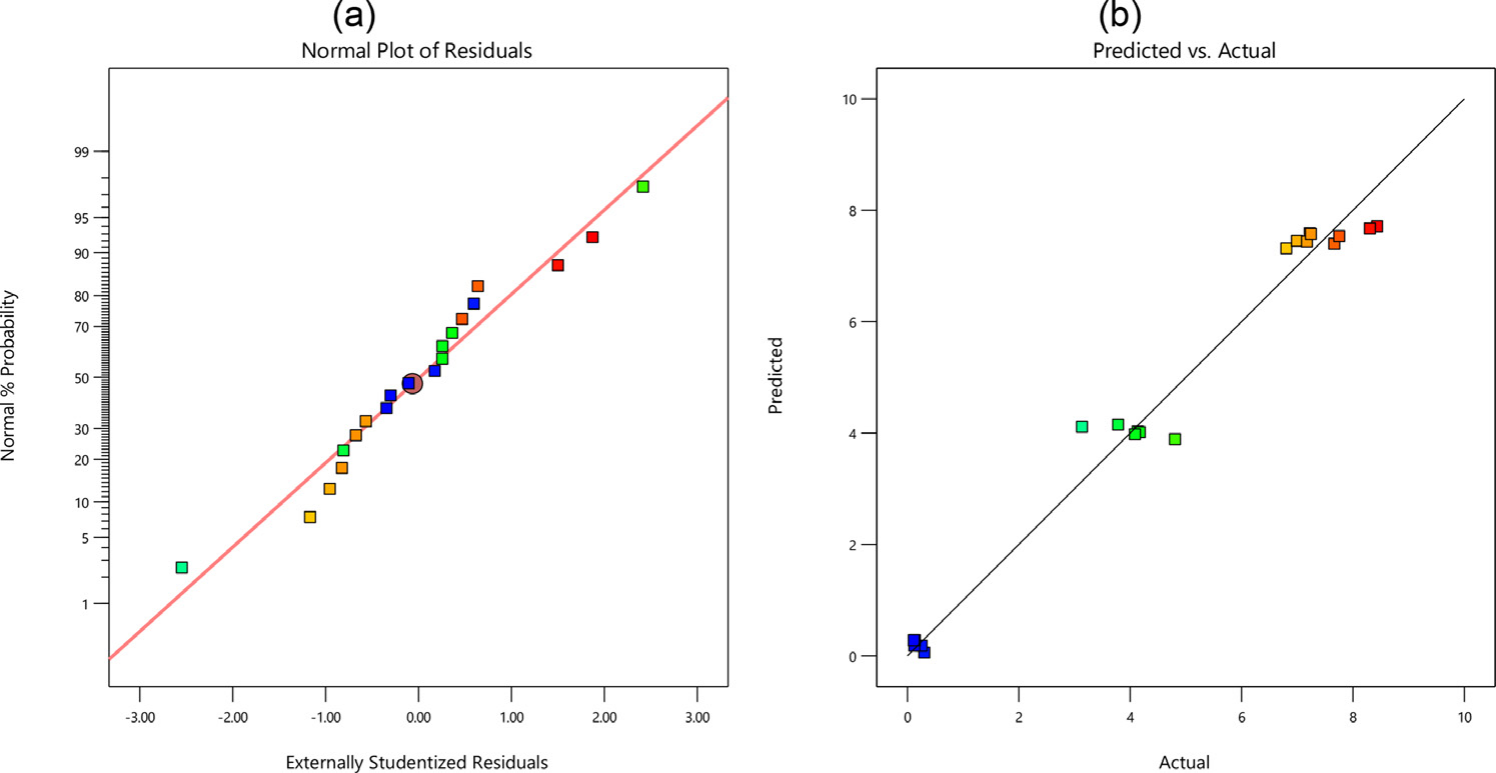
The elastic module: (a) the normal plot of residuals and (b) the variations of the predicted and experimental data; Note: Color points are from blue (0.12) to red (8.44)

**Fig. 7:**
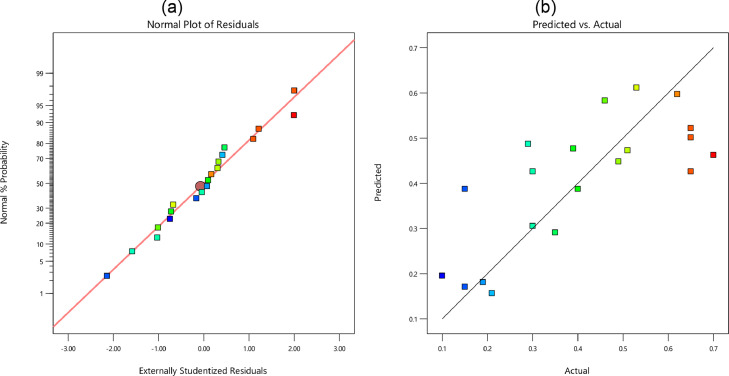
The friction coefficient: (a) the normal plot of residuals and (b) the variations of the predicted and experimental data; Note: Color points are from blue (0.1) to red (0.7)

**Fig. 8:**
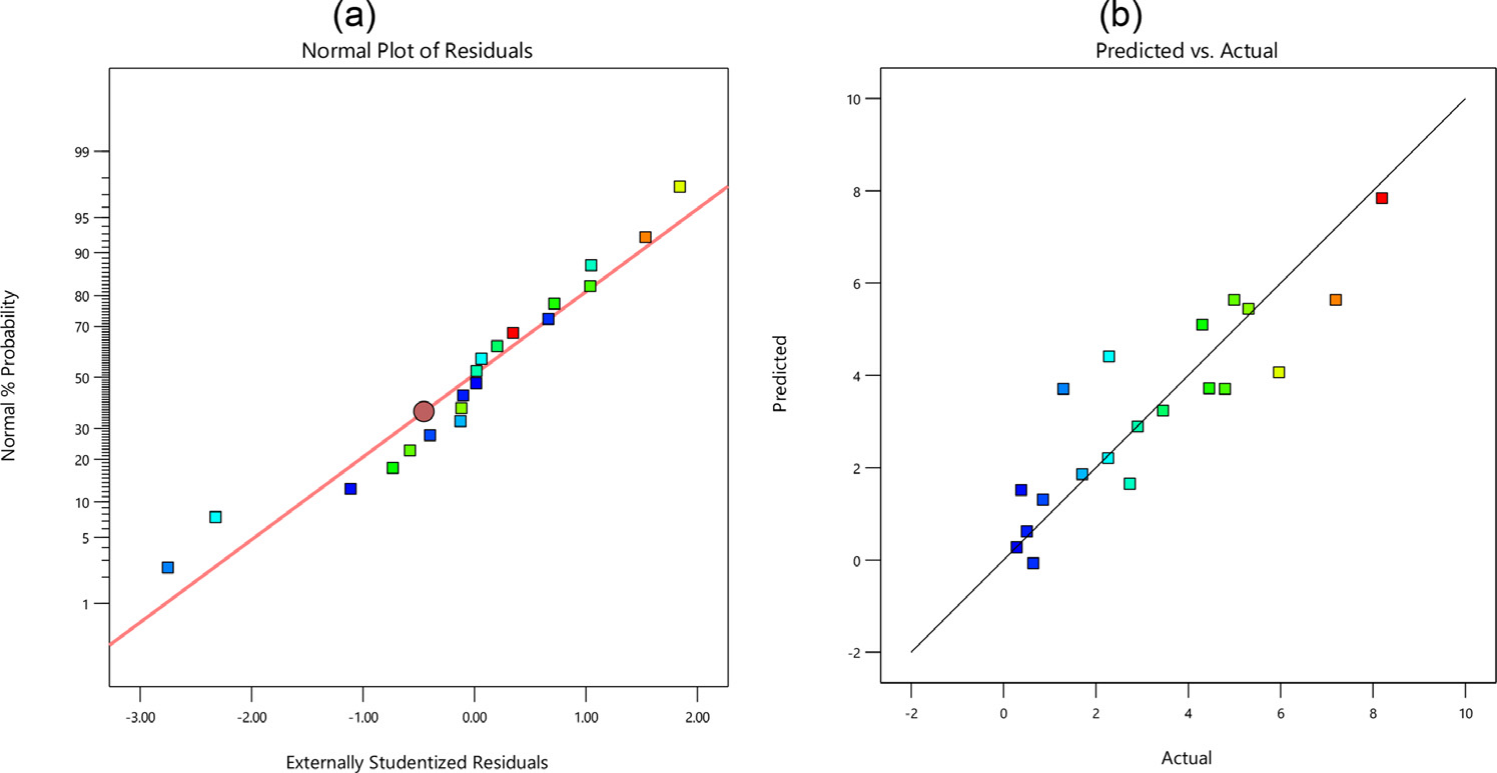
The wear rate: (a) the normal plot of residuals and (b) the variations of the predicted and experimental data; Note: Color points are from blue (0.29) to red (8.20)

**Fig. 9:**
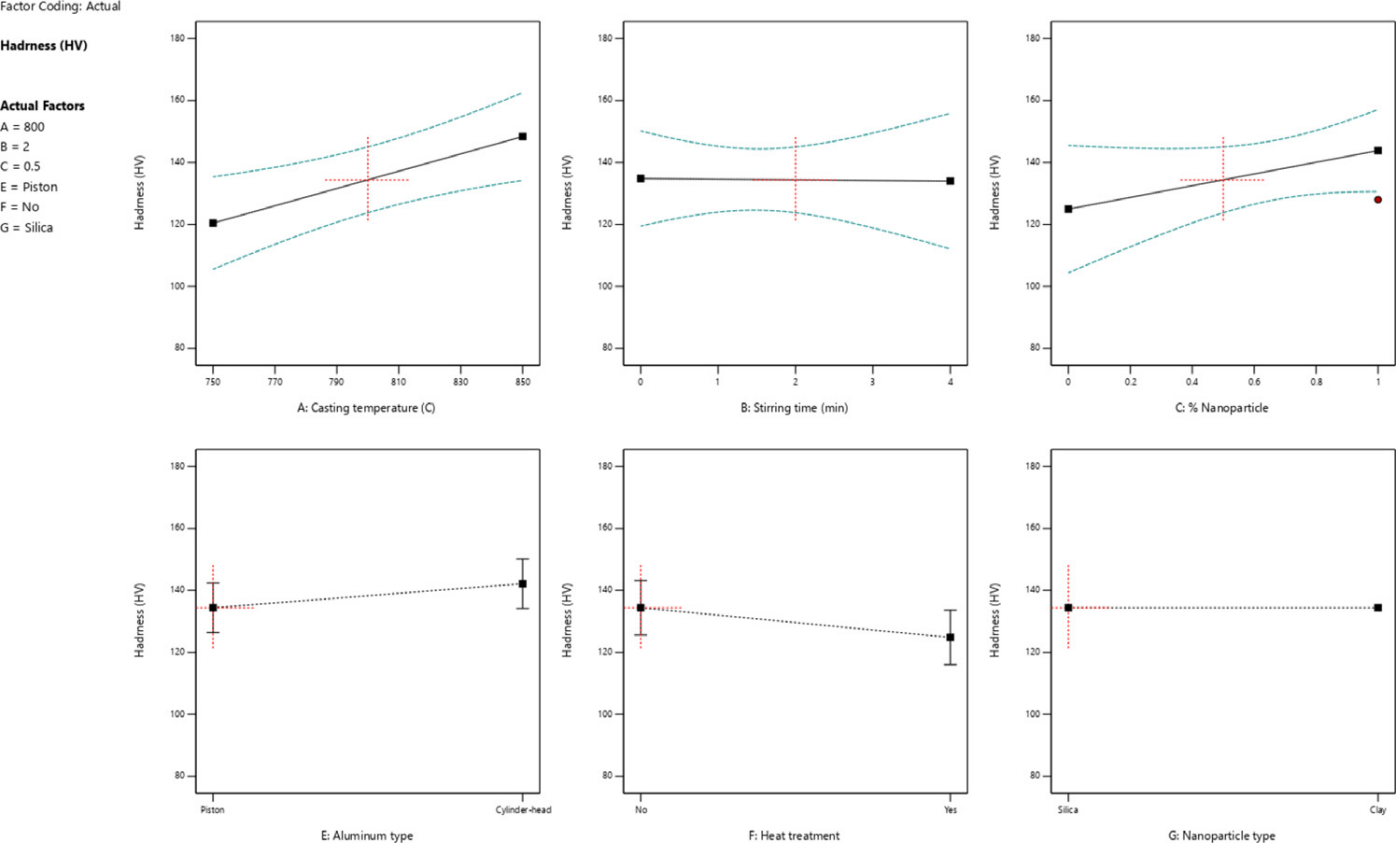
The variable influences on the hardness; Note: The nanoparticle type was ignored

**Fig. 10:**
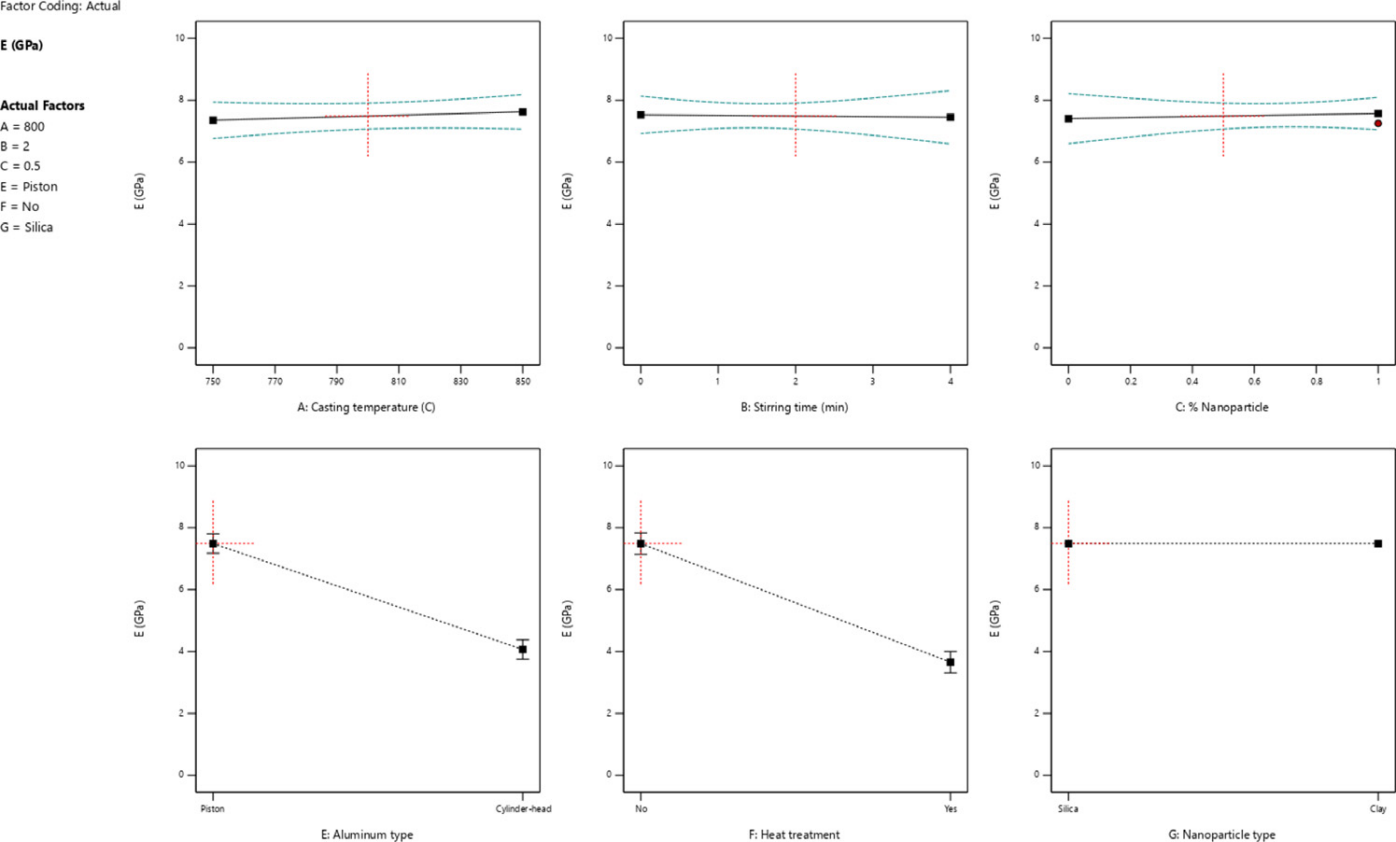
The variable influences on the elastic module; Note: The nanoparticle type was ignored

**Fig. 11:**
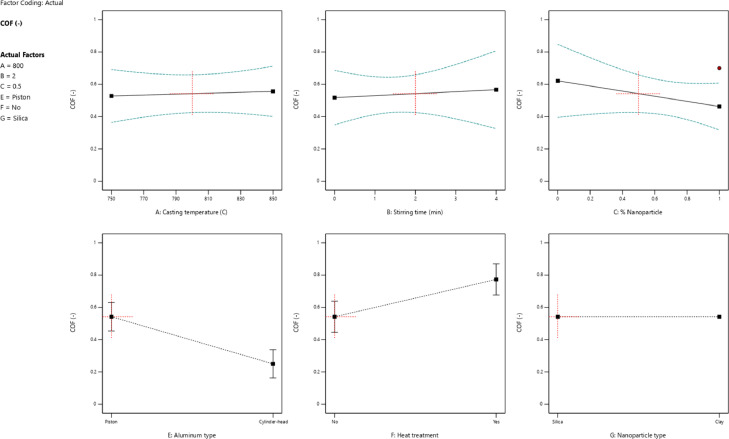
The variable influences on the friction coefficient; Note: The nanoparticle type was ignored

**Fig. 12:**
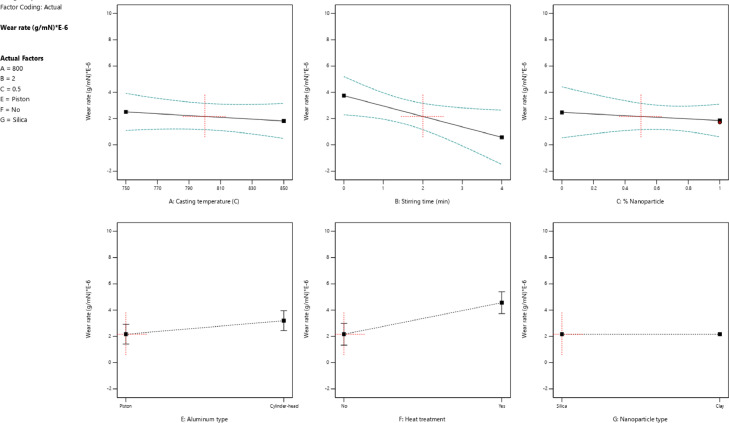
The variable influences on the wear rate; Note: The nanoparticle type was ignored

In Figs. 5, 6, 7, and 8, the probability of residual values is illustrated for different outputs. Besides, the scatter-band of the predicted and experimental values is also presented in these figures. Based on these results, the scatter-band of the wear rate was narrower than others. In Figs. 9, 10, 11, and 12, the variable influences are demonstrated for the hardness, *E*, COF, and the wear rate, in which the trend behavior of inputs is clear as increasing or decreasing ones. Notably as mentioned before, the nanoparticle type was ignored in the regression model.

**Fig. 13:**
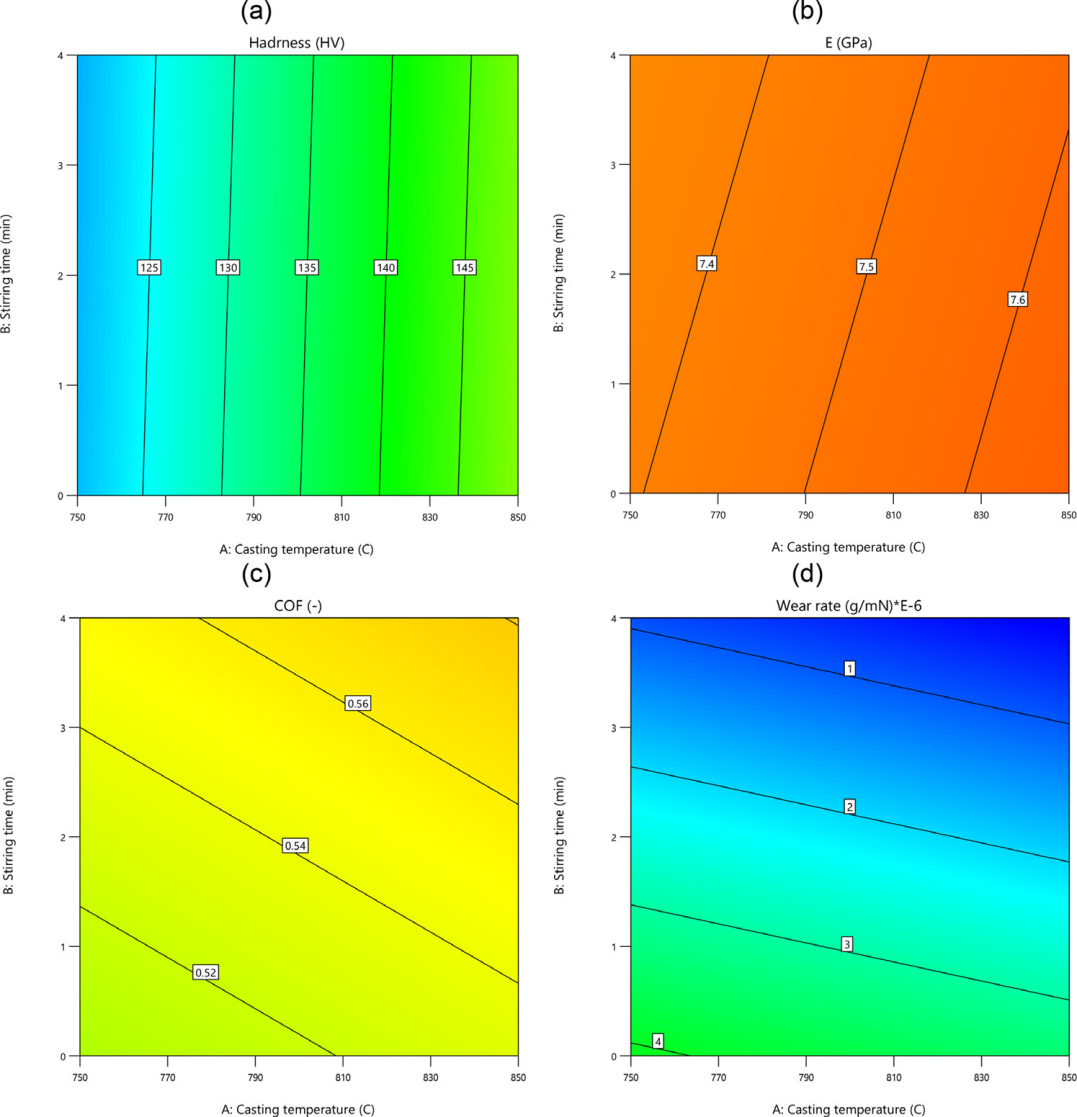
The contour plots of the stirring time and the casting temperature: (a) the hardness, (b) the elastic module, (c) the friction coefficient, and (d) the wear rate

**Fig. 14:**
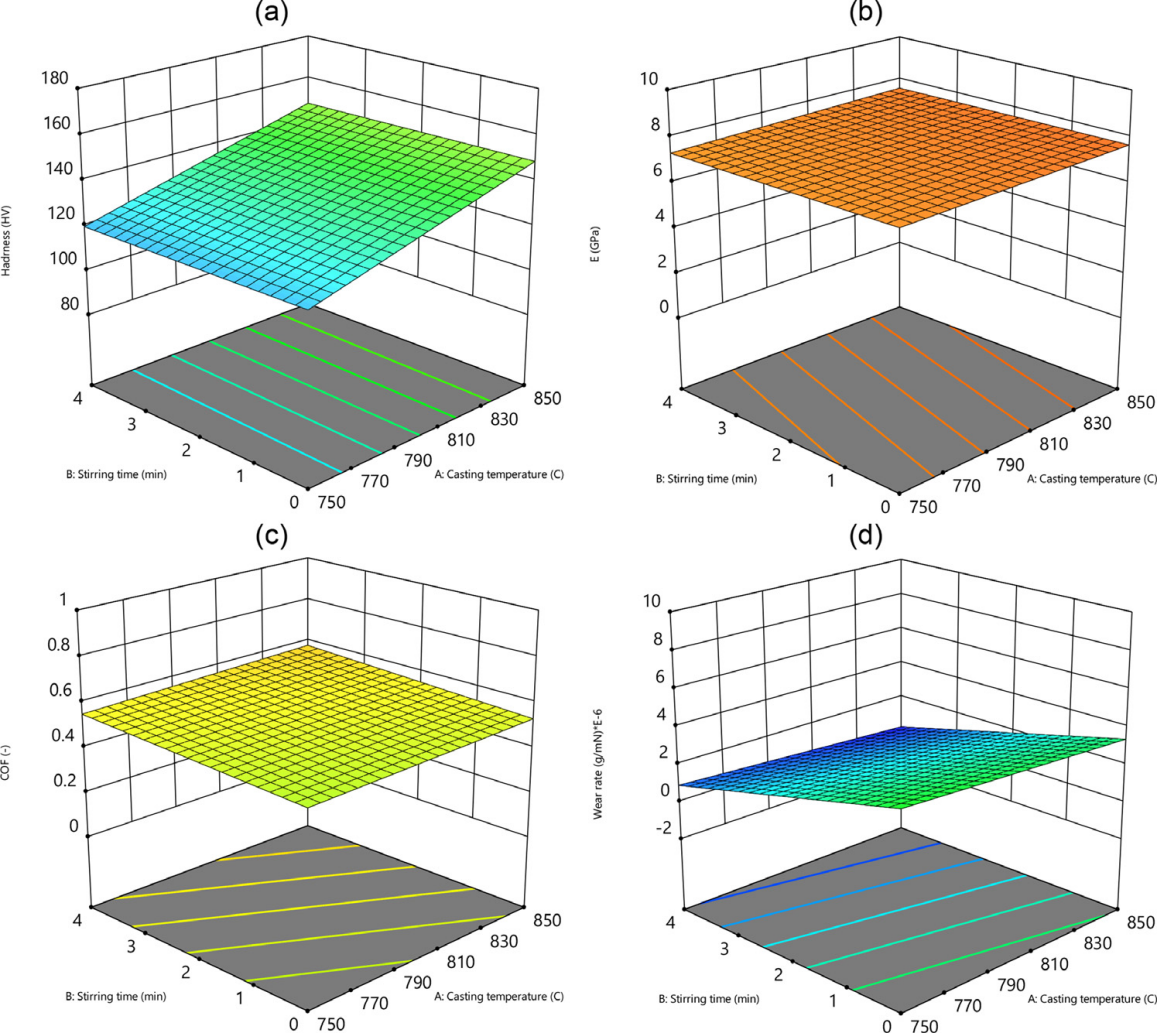
The surface plots of the stirring time and the casting temperature: (a) the hardness, (b) the elastic module, (c) the friction coefficient, and (d) the wear rate

Lastly, the contour plots and the surface plots of outputs are presented in Fig. 13, Fig. 14, respectively for the inputs of the casting temperature and the stirring time.

## 2 Materials and Experimental Design

The piston aluminum alloy was reinforced by silica (SiO_2_) nanoparticles. The chemical composition of this alloy was 12.50% Si, 2.40% Cu, 2.20% Ni, 0.74% Mg, 0.41%Fe, and balanced with Al. However, the cylinder-head aluminum alloy was strengthened by clay nanoparticles. The chemical composition of such alloy was 7.20% Si, 3.50% Cu, 0.42% Fe, 0.37% Mn, 0.26% Mg, and balances with Al. The mean size of the silica and clay nanoparticles was about 25 and 40 nm, respectively. The percentage of both nanoparticles was about 1%. More details of utilized nanoparticles were found in other research [2,3].

To manufacture various specimens, a vertical tube furnace was used to melt aluminum alloy. Three casting temperatures of 750, 800, and 850 °C were chosen.It was notable that the casting temperature was as the same as the stirring temperature. In addition, the stirring times were 2 and 4 for nanocomposite manufacturing. A stirrer made from hot-work tool steel was utilized for stirring action in the casting process. For deoxidizing of melt during the casting process, argon gas was purged into the aluminum melt. The ageing heat treatment was as follows, solutionizing at 490 °C for 5 hrs, water-quenching, and ageing at 200 °C for 2 hrs.

To study the tribological behavior of specimens, a pin-on-disk tribometer was applied. Wear tests were performed based on ASTM: G99-95A at a temperature of 20-25 °C [4]. The wear distance for all specimens was 500 m. The applied normal load was 7 N. In wear testing, the rotation speed was 200 rpm. A steel pin with a hardness value of 778 HV and a diameter value of 3 mm was subjected to surfaces of specimens.

A Vickers hardness tester (Ericsson E0014 model) was also used for measuring the hardness of specimens based on ASTM: E92 [5]. For each specimen, five indentations were used and a mean measurement was reported.

Compression test samples were prepared based on ASTM D695 [6] with a universal testing machine (Instron-1195). The ratio of L/D for all specimens was kept at 1. The compression speed was about 1 mm min^−1^. This test was performed until macroscopic damages were formed on specimens.

Finally, all inputs are defined in Table 5 with the type and minimum and maximum values.

**Table 5:** The inputs for the sensitivity analysis of the experimental data.

Factor	Name	Units	Type	Minimum	Maximum	Level	Mean	Std. Dev.
A	Casting temperature	(C)	Numeric	750	850	3	800.00	36.63
B	Stirring time	(min)	Numeric	0	4	3	2.00	1.65
C	Percentage of Nanoparticle	(%)	Numeric	0	1	2	0.70	0.47
E	Aluminum type	(-)	Categoric	Piston	Cylinder-head	2	-	-
F	Heat treatment	(-)	Categoric	No	Yes	2	-	-
G	Nanoparticle type	(-)	Categoric	Silica	Clay	2	-	-

## Ethics Statements

It is not applicable to this analyzed dataset.

## CRediT Author Statement

**Mahboobeh Azadi:** Data curation, Conceptualization, Methodology, Investigation, Validation, Writing – original draft preparation, Writing – review & editing, Supervision; **Mohammad Azadi:** Conceptualization, Software, Methodology, Visualization, Investigation Writing – original draft preparation, Writing – review & editing, Supervision.

## Declaration of Competing Interest

The authors declare that there is no known competing financial interests or personal relationships for this work.

## Data Availability

Tribological Data for Reinforced Aluminum-Silicon Alloys (Original data) (Mendeley Data).
